# Sampling frequency affects estimates of annual nitrous oxide fluxes

**DOI:** 10.1038/srep15912

**Published:** 2015-11-02

**Authors:** L. Barton, B. Wolf, D. Rowlings, C. Scheer, R. Kiese, P. Grace, K. Stefanova, K. Butterbach-Bahl

**Affiliations:** 1 Soil Biology and Molecular Ecology Group, School of Earth & Environment (M087), UWA Institute of Agriculture, Faculty of Sciences, The University of Western Australia, 35 Stirling Highway, Crawley, Western Australia 6009, Australia.; 2 Karlsruhe Institute of Technology, Institute for Meteorology and Climate Research Atmospheric Environmental Research (IMK-IFU), Kreuzeckbahnstr. 19, 82467 Garmisch-Partenkirchen, Germany.; 3 Institute for Future Environments, Queensland University of Technology, 2 George Street, Brisbane, Queensland 4000, Australia.; 4 UWA Institute of Agriculture (M089), The University of Western Australia, 35 Stirling Highway, Crawley, Western Australia 6009, Australia.; 5 International Livestock Research Institute (ILRI), Nairobi, Kenya

## Abstract

Quantifying nitrous oxide (N_2_O) fluxes, a potent greenhouse gas, from soils is necessary to improve our knowledge of terrestrial N_2_O losses. Developing universal sampling frequencies for calculating annual N_2_O fluxes is difficult, as fluxes are renowned for their high temporal variability. We demonstrate daily sampling was largely required to achieve annual N_2_O fluxes within 10% of the ‘best’ estimate for 28 annual datasets collected from three continents—Australia, Europe and Asia. Decreasing the regularity of measurements either under- or overestimated annual N_2_O fluxes, with a maximum overestimation of 935%. Measurement frequency was lowered using a sampling strategy based on environmental factors known to affect temporal variability, but still required sampling more than once a week. Consequently, uncertainty in current global terrestrial N_2_O budgets associated with the upscaling of field-based datasets can be decreased significantly using adequate sampling frequencies.

Scientists have been quantifying soil nitrous oxide (N_2_O) fluxes in the field for at least 60 years^1^. Interest in soil N_2_O fluxes originated from a desire to better understand the fate of soil nitrogen^2^,^3^. Efforts to measure soil N_2_O fluxes were further increased when N_2_O was recognized as a potent greenhouse gas (GHG) that also plays a significant role in the depletion of stratospheric ozone^4^,^5^. Quantifying annual soil N_2_O emissions, the dominating source for atmospheric N_2_O, has consequently become a priority for signatory countries to the UN Framework Convention on Climate Change, who are required to present national GHG inventories on an annual basis to the Conference of Parties ( http://unfccc.int).

Quantifying annual N_2_O fluxes from soils is challenging. Fluxes vary spatially, and differ from day-to-day (and within the day) in response to multiple factors that regulate N_2_O production, consumption and emission^6^. Manual (static) chambers are currently the most widely used technique for quantifying soil N_2_O fluxes. Chamber flux measurements are short-term (e.g., hourly), repeated usually in intervals of days to weeks, and are in turn integrated across time to finally calculate an annual losses. However, fluxes estimated using static chambers can be adversely affected by inadequate mixing of the headspace air, pressure changes, and increases in headspace gas concentration in response to changes to the natural concentration gradient between the soil and atmosphere^7^. Despite these documented short-comings, this approach is favored for N_2_O measurements as fluxes can be orders of magnitude smaller than CO_2_ fluxes and the accumulation of gas in the headspace leads to best results with respect to flux detection limit compared to, for instance, dynamic chamber or micrometeorological techniques^8^. Furthermore manual chambers are simple to use, relatively inexpensive, and can be deployed in small experimental plots so the effect of multiple treatments on soil N_2_O fluxes can be investigated simultaneously^6^. Temporal coverage is typically limited to weekly, bi-weekly or monthly measurements when using manual chambers^9^. However, manual chambers are likely to underestimate annual N_2_O fluxes if the frequency of measurements does not adequately characterize N_2_O emissions during the year, in particular peak emissions following N fertilizer applications, irrigation, soil re-wetting or spring-thaw events, which may contribute up to 70% of the total annual flux^10^,^11^,^12^.

Guidelines for sampling frequency to estimate annual N_2_O fluxes using manual chambers are not well defined for all land-uses and environments. Instead the approach often taken comes down to an “educated guess” and resource availability^13^. A number of studies have investigated the influence of sampling frequency on cumulative N_2_O fluxes, however with the exception of Liu *et al.*^14^ these studies have mainly used short-term N_2_O flux data sets (<1 year), and have been confined to a single study site in an agricultural setting^9^,^15^,^16^,^17^,^18^. These short-term studies have demonstrated that high frequency measurements should coincide with management practices likely to increase N_2_O fluxes, with less frequent measurements during the intervening periods. Yet, the effect of sampling frequency on annual N_2_O flux estimates requires investigation across a broader range of land-uses and climates.

The introduction of automated chambers has enabled researchers to better characterize temporal variation in N_2_O fluxes^6^. Although this technology is expensive and not available to all researchers, it does provide a unique opportunity to better assess how sampling frequency affects estimates of annual N_2_O fluxes. Such an analysis is particularly beneficial to those new to measuring *in situ* N_2_O fluxes from land and to those planning to investigate a previously unstudied land-use. Consequently, the objective of the following study was to investigate the effect of sampling frequency on estimates of annual soil N_2_O fluxes using 28 published datasets of subdaily N_2_O fluxes from a variety of different terrestrial ecosystems across the globe.

## Results

**Figure 1:**
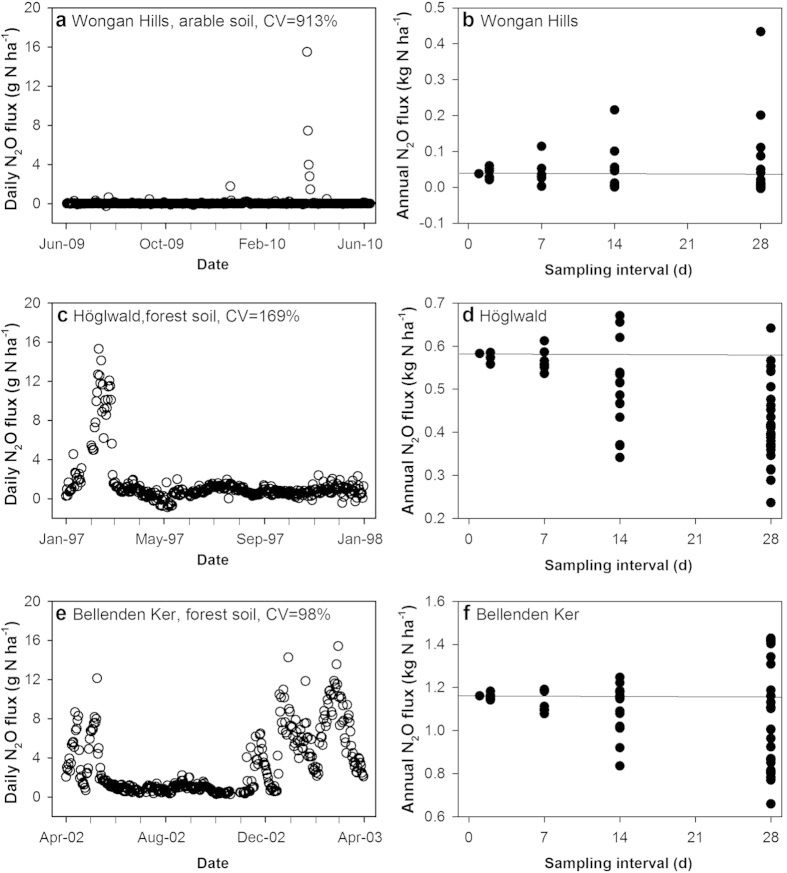
Daily N_2_O fluxes and the influence of sampling frequency on annual N_2_O fluxes. The daily N_2_O flux (**a,c,e**) for the three data sets shown have varying coefficients of variation (CV), which influences the effect of sampling frequency on annual N_2_O fluxes (**b,d,f**). See Table 4 for description of sampling intervals.

Annual N_2_O fluxes calculated from the average daily fluxes, which is used here as the reference annual flux, varied from 0.03 kg N_2_O-N ha^−1^ yr^−1^ to 8.1 kg N_2_O-N ha^−1^ yr^−1^ (Table 1). The smallest annual flux was recorded for a sandy soil cropped to a grain legume in a semiarid environment^11^, while the greatest was from a loam soil cropped to tree fruit in a subtropical climate^19^. Daily N_2_O fluxes were highly variable within each dataset, but more so for some; the coefficient of variation (CV) of the mean daily N_2_O flux ranged from 78% for a subtropical rainforest to 913% for a semiarid soil planted to a grain legume. The variation in daily means was not related to the magnitude of the annual N_2_O flux (Table 1), but instead reflected the episodic nature of the daily fluxes for a particular study site (Fig. 1). We subsequently classified the data sets as having either moderate (CV > 50–100%), high (CV > 100–200%) or extreme (CV > 200%) ‘episodicity’ based on the CV of the mean daily flux (Table 2).

**Figure 2:**
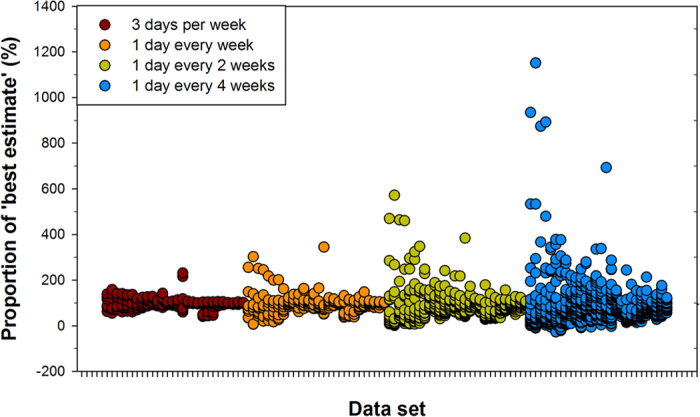
The proportion (%) of the ‘best estimate’ annual N_2_O flux estimated by each sampling frequency. For each dataset (28), the average annual flux estimate (calculated from replicate chambers) for each sampling frequency (and each permutation, Table 4) was compared to the ‘best estimate’ flux calculated from the average daily fluxes (expressed as a %). The ‘best estimate’ was calculated using all daily fluxes. For each sampling frequency, the datasets are presented in the same order (from left to right in the above Figure) as that listed in Supplementary Table 1. Specific values for each dataset are listed in Supplementary Table 1.

**Figure 3:**
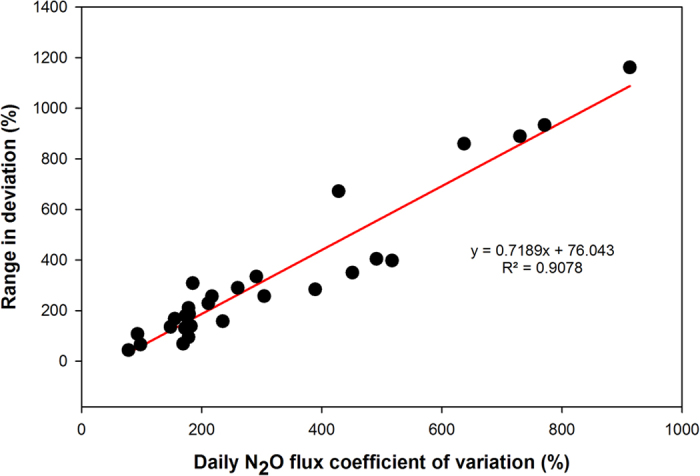
Relationship between the coefficient of variation of the daily N_2_O flux and the deviation (range) from the ‘best estimate’ annual N_2_O flux. For each dataset (28; represented as single point in the above Figure), the range in deviation was determined after comparing the annual N_2_O fluxes calculated from a sample interval of 4-weekly (every 28 days) with the ‘best estimate’ for each permutation (Table 4). The ‘best estimate’ was calculated using all daily fluxes. Specific values for each dataset are listed in Supplementary Table 1.

Increasing the interval between sampling days increased the variance in the estimated annual N_2_O fluxes, and hence decreased the accuracy of the estimate (Fig. 1). As the sampling frequency decreased, the deviation from the ‘best estimate’, or expected value obtained using all daily fluxes, increased and caused annual losses to be either over- or underestimated (Fig. 2; Supplementary Table 1). Across all sites and sampling frequencies (n = 1568), 22% and 58% of annual emission values were more than 10% higher or lower, respectively, than the ‘best estimate’ annual flux. The extent that decreased sampling frequency increased the deviation from the reference annual N_2_O flux appeared to be largely related to the variability, or coefficient of variation, of the daily fluxes (Fig. 3). The greater the variation in daily N_2_O within a dataset, the greater the impact of decreasing the sampling frequency had on the accuracy of the estimated annual flux. For example, for a tropical rainforest (Bellenden Kerr) with a daily N_2_O flux CV of 98%, sampling every 28 days resulted in an annual N_2_O flux that was up to 1.2 times greater than the best estimate; whereas for cropped soil in a semiarid region with a daily N_2_O flux CV of 913%, sampling every 28 days resulted in an annual N_2_O flux that was up to 12 times greater than the best estimate (Fig. 1; Supplementary Table 1).

**Figure 4:**
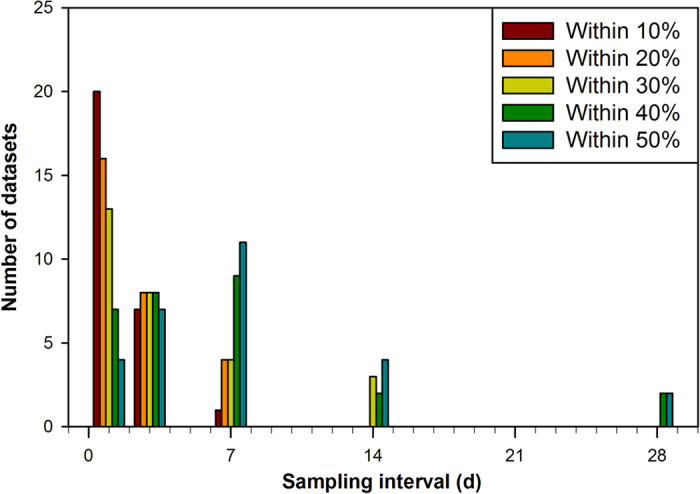
Impact of sampling interval on estimating annual N_2_O fluxes at a given accuracy. The number of datasets obtaining annual N_2_O fluxes at a given accuracy are listed as a function of sampling interval. Specific values for each datasets are listed in Supplementary Table 1.

The minimum sampling frequency required to robustly estimate an annual N_2_O flux varied depending upon the ‘episodicity’ of the dataset and the required accuracy (Fig. 2; Table 2; Supplementary Table 1). Twenty, or 74%, of the datasets required daily sampling to achieve an annual N_2_O flux value within 10% of the best estimate (Fig. 4). In only one case (tropical rainforest, Bellenden Ker), and when the daily N_2_O flux CV was relatively low (98%), did weekly sampling result in annual N_2_O flux within 10% of the best estimate. Generally speaking, highly or extremely episodic data sets (CV > 100%) required sampling either daily or 3 days a week (Table 2). Lowering the desired accuracy decreased the required frequency of sampling, however 89% of the data sets still needed to be sampled at least weekly to achieve ±30% accuracy (Fig. 4). Lowering the level of accuracy to ±40% meant two datasets could be sampled once every 4 weeks (Fig. 4).

## Discussion

Nitrous oxide emissions need to be measured daily to accurately determine annual N_2_O flux in environments where data has not previously been collated. Measuring N_2_O fluxes on a daily basis ensured that annual N_2_O fluxes were estimated within 10% of the expected value for all datasets in the present study. Although a similar result could be achieved in some instances (25%, or 7 datasets) by sampling 3 days a week, this still represents a highly regularly sampling regime. Our findings are consistent with others who have investigated the effects of sampling frequency on estimates of annual N_2_O fluxes. For example, a relatively frequent sampling regime (once every 2 to 3 days) was required to estimate cumulative losses within 10% of the expected cumulative loss from N-fertilized crops in China and the United States of America^9^,^14^. Our findings further confirm the importance of deploying automated chamber systems when determining annual N_2_O fluxes in previously unstudied environments, and when the drivers of temporal variability are not well understood.

The frequency of sampling required to accurately calculate an annual N_2_O flux will depend on the episodic nature of the N_2_O flux at the study site of interest, rather than the magnitude of the annual flux. This was particularly well demonstrated by studies conducted in semiarid environments of Australia and Inner Mongolia, where relatively low annual N_2_O losses ( ≤ 0.21 kg N_2_O-N ha^−1^ yr^−1^ ) resulted from a limited number of elevated daily N_2_O fluxes during the year^11^,^12^. For example in a cropped soil in south-western Australia, 75 to 85% of the annual fluxes were attributed to isolated, short-lived summer rainfall events^11^. Understanding the underlying temporal variability of daily N_2_O fluxes is therefore likely to improve the efficacy of sampling regimes.

Sampling efficacy for determining annual N_2_O fluxes may be improved, and the regularity of sampling decreased, if N_2_O flux responses can be anticipated. This may occur if previous research has been conducted in a similar environment, or if preliminary work is undertaken to assess the temporal variability of N_2_O fluxes. In either case, refining the sampling regime will require some underlying understanding of temporal variation in the N_2_O flux and its regulation. Using this approach, we estimated annual N_2_O fluxes for three of our datasets (Fig. 1) based on the authors’ informed understanding of the factors driving daily losses. While we found the annual N_2_O fluxes estimated by the authors’ did not vary statistically from the ‘best’ estimate calculated using all daily fluxes, the informed sampling approach still required sampling to occur every 2 to 6 days depending on the dataset (Table 3). A number of short-term studies have also devised strategies for characterizing N_2_O fluxes in response to N inputs to cropping and grazed soils setting^9^,^15^,^16^,^17^,^18^. Notably, various authors used automated chambers to develop a sampling regime for measuring N_2_O emissions from temperate grasslands in response to ruminant urine deposition^16^ in New Zealand, N fertilized potato fields in Europe^18^, and rainfed cereal crops in subtropical Australia^17^; agricultural land use not captured in the present study. Interestingly, some of these authors recommended weekly sampling (with a higher frequency following anticipated N_2_O events), which is less frequent than our analysis would recommend for agricultural and non-agricultural study sites in the present study.

The uncertainty of current global N_2_O estimates maybe partly attributed to the sampling frequency of the datasets selected for inclusion in the analysis. Modelling of global soil N_2_O emissions has been largely derived from manual chambers measurements covering more than 300 days in a year^20^. However, less than a third of the 464 studies included in the metadata analysis by Stehfest and Bouwman^20^ measured N_2_O on at least a daily basis, with 50% of the data used collected 3 days a week, or less than weekly. Given the influence of sampling frequency on annual N_2_O fluxes in the present paper, it is likely that current global N_2_O values have not been accurately captured. Instead, we recommend that revision of global estimates using high frequency measurements (at least daily) or an ‘informed’ sampling approach for at least a year.

Finally, we recommend data from automated chambers should be continuously used to build on existing guidelines for use of manual chambers^21^. While the present study included and discussed a large number of datasets from a variety of climates, soils and land uses, there were a number of environments not represented. For example, grazed soils outside temperate climate, a broader range of horticultural soils, and non-agricultural soils in semiarid environments. We therefore encourage researchers utilizing automated chamber systems to determine annual N_2_O fluxes from soils, to in turn also utilize the data to investigate the impacts of sampling frequency on these losses.

## Methods

### Study sites

The meta-analysis included datasets from published research studies, and where N_2_O fluxes had been measured on a subdaily basis for approximately one year using automated chambers. Annual data sets were sourced from measurements in Australia, Germany, and Inner Mongolia, representing a variety of climates, soil types and land uses (Table 1). Climates ranged from semiarid (including a Mediterranean-type climate) to tropical, soil textures varied from sands to heavy clays, while land use included various agricultural production and forest systems. A number of study sites also included different treatments (Table 1). Consequently our meta-analysis included 28 sub-daily N_2_O datasets.

### Automated chamber system

Nitrous oxide fluxes were measured at each study location using soil chambers connected to a fully automated system that enabled *in situ* determination of N_2_O fluxes. Details of the design and operation of the automated gas sampling systems have been described by Breuer *et al.*^22^ and Kiese *et al.*^23^. Briefly, the various systems consisted of a gas chromatograph (e.g., Texas Instruments, SRI 8610C) equipped with an electron capture detector (ECD) for N_2_O analysis, an automated sampling unit for collecting and distributing gas samples, and a series of chambers (three to five replicates depending on the study site). Chambers (0.5 m × 0.5 m or 0.7 m × 0.7 m) were placed on metal bases inserted into the ground (0.05–0.1 m), and fitted with a top (0.15 m or 0.3 m in height) that could be automatically opened and closed by means of pneumatic actuators. The height of the chambers was progressively increased to accommodate crop growth at some study sites, with a maximum height of 0.95 m. Furthermore, in some instances the chambers were programmed to open if the air temperature in the chamber exceeded a set value, or if rain fell while the chambers were closed. The automated gas sampling unit enabled N_2_O to be monitored continuously, providing up to eight (hourly) emission rates per day. Specific N_2_O measurement details for each study site are described in the associated published papers (Table 1).

### Evaluating sample frequency effects

The effect of sampling frequency on estimates of annual N_2_O-N fluxes was assessed using a modified jackknife technique^24^,^25^. Average daily flux measurements were calculated for each replicate chamber in each dataset from the sub-daily flux measurements as we did not consistently observe diurnal flux variations at each location. Each site’s daily flux population was subsequently subsampled daily, three times per week, weekly, bi-weekly and 4-weekly, and for each permutation of the time interval, for each dataset (Table 4). There were 7 to 28 jackknifed populations depending on the sampling frequency (Table 4). Estimates of annual N_2_O-N flux for a given chamber, site and frequency permutation were then calculated by linear interpolation and integration of daily fluxes with time. Missing daily N_2_O flux data was not replaced. The average annual flux estimate (calculated from replicate chambers) from each sampling frequency, and for each of the dataset, was then compared to the ‘best estimate’ annual flux calculated from the average daily fluxes (expressed as a %) so as to assess the accuracy of each of the sampling frequencies. An annual flux determined using an informed sampling regime (based on the authors’ understanding of the factors driving daily N_2_O fluxes) was also compared with the ‘best estimate’ annual flux using a general analysis of variance (Genstat for Windows, 14^th^ Edition, VSN International).

## Additional Information

**How to cite this article**: Barton, L. *et al.* Sampling frequency affects estimates of annual nitrous oxide fluxes. *Sci. Rep.* **5**, 15912; doi: 10.1038/srep15912 (2015).

## Supplementary Material

Supplementary Information

## Figures and Tables

**Table 1:** Summary of data sets used to assess the effect of sampling frequency on estimated annual N_2_O fluxes.

Location†, Year	Climate	Rainfall‡ (mm yr^−1^)	Soil C§ (g kg^−1^)	Soil texture§	Land use	Annual datasets	Study period (days)	Annual flux (kg N_2_O-N ha^−1^yr^−1^)	Daily flux CV (%)	Reference
Wongan Hills, Australia. 2009–2011	Semiarid	374	10	Sand	Grain crop, rainfed,+/− lime,+/− N fertilizer	8	364–371	0.03–0.07	380–913	Barton *et al.*^11^
Cunderdin, Australia. 2005–2009	Semiarid	368	9.8	Sand	Grain crop, rainfed,+/- N fertilizer	8	337–379	0.08–0.16	173–428	Barton *et al.*^10^ Li *et al.*^26^ Barton *et al.*^27^ Barton *et al.*^28^
Xilin, Inner Mongolia. 2007–2008	Semiarid, cool temperate	335	26	Sandy loam	Steppe grassland, not grazed	1	365	0.21	260	Wolf *et al.*^12^
Höglwald, Germany. 1996–1997	Temperate	850	22	Silty clay	Spruce and beech forest (plantation)	2	365	0.58–2.46	169–179	Papen & Butterbach-Bahl (1999)^29^ Wu *et al.*^30^
Kingsthorpe, Australia. 2009–2010	Subtropical	630	15	Clay	Wheat-cotton crop, irrigated, N fertilizer	3	334	2.61–2.93	181–235	Scheer *et al.*^31^ Scheer *et al.*^32^
Mooloolah Valley, Australia. 2007–2009	Subtropical	1709	28	Loam	Pasture, mowed, not grazed	2	365	1.16–2.12	155–172	Rowlings *et al.*^33^
Mooloolah Valley, Australia.2008–2009	Subtropical	1709	35	Loam, silt loam	Rainforest (notophyll vine)	1	365	0.48	78	Rowlings *et at.*^34^
Mooloolah Valley, Australia. 2007–2009	Subtropical	1709	27	Loam	Tree crop (lychee)	2	365	1.68–8.12	93	Rowlings *et at.*^19^
Bellenden Ker, Australia. 2001–2002	Tropical	4360	31	Sandy loam	Rainforest (mesopyll vine)	1	365	1.16	98	Kiese *et al.*^23^

^†^Cunderdin, 31°36′S, 117°13′E; Wongan Hills, 30°89′S, 116°72′E; Höglwald 48°30′N, 11°10′E; Xilin 43° 33′ N, 116° 42.3′ E; Bellenden Ker, 17°16′S, 145°54′E; Kingsthorpe, 27°30′S, 151°46′E; Mooloolah Valley 26°75′ S, 152°93′ E

^‡^Long-term average value

^§^Surface soil (e.g., 0–15cm).

**Table 2:** The relationship between the ‘episodicity’ of each study location and the minimum sampling frequency needed to meet a given accuracy.

Location		Minimum sampling frequency	
	Episodicity†	10% accuracy	0% accuracy
Wongan Hills, Australia.	Extreme	Daily	Daily to 3 days a week
Cunderdin, Australia.	High to Extreme	Daily or 3 days a week	Daily to weekly
Xilin, Inner Mongolia.	Extreme	3 days a week	Weekly
Höglwald, Germany.	High	3 days a week	Weekly to bi-weekly
Kingsthorpe, Australia.	High to Extreme	Daily	Daily
Mooloolah Valley, (Pasture) Australia.	High	Daily to 3 days a week	3 days a week to weekly
Mooloolah Valley, (Rainforest) Australia.	Moderate	3 days a week	Bi-weekly
Mooloolah Valley, (Tree crop) Australia.	Moderate	3 days a week	Weekly
Bellenden Ker, Australia.	Moderate	Weekly	Bi-weekly

^†^Episodicity determined using coefficient of the mean daily flux (Table 1). Moderate, CV > 50–100%; high, CV > 100–200%; extreme, CV > 200%.

**Table 3:** Annual N_2_O fluxes for three contrasting study sites estimated using either an informed sampling strategy or from daily measurements.

Location†	Best estimate	Informed sampling regime‡	
	Annual flux (kg N_2_O-N ha^−1^ yr^−1^)	Annual flux (kg N_2_O-N ha^−1^ yr^−1^)	No. measurements
Wongan Hills, Australia.	0.04 (0.0)^a^	0.03 (0.0)^a^	60
Höglwald, Germany.	0.58 (0.1)^a^	0.64 (0.1)^a^	83
Bellenden Ker, Australia.	1.16 (0.1)^a^	1.35 (0.2)^a^	156

Values represent means (and standard errors) of three to five replicates depending on the study site. Values followed by the same letter in the same row are not statistically different (*P *< 0.05).

^†^For further details see Table 1. Corresponds to data set numbers 2 (Wongan Hills), 19 (Höglwald) and 28 (Bellenden Ker) in Supplementary Table 1.

^‡^Informed sampling strategy for each location: Wongan Hills, N_2_O fluxes measured daily for five consecutive days when daily rainfall > 5 mm rainfall (December–June) or > 10 mm rainfall (July–November); Höglwald, N_2_O fluxes measured daily for seven days consecutive day when air temperature between −0.7 and 0.7 °C or if daily rainfall > 15 mm; Bellenden Ker, N_2_O fluxes measured daily for six days consecutive day when daily rainfall > 15 mm.

**Table 4:** Description of sampling frequencies.

Sampling frequency	Sampling interval (days)	Permutations	Examples
Daily	1	1	Sunday
			Monday
			Tuesday *etc*
3 days a week	2	7	Sunday-Tuesday-Thursday
			Monday-Wednesday-Friday
			Tuesday-Thursday-Saturday *etc*
1 day a week (weekly)	7	7	Every Sunday
			Every Monday
			Every Tuesday *etc*
1 day every 2 weeks (bi-weekly)	14	14	Every Sunday (week 1 of 2)
			Every Sunday (week 2 of 2)
			Every Monday (week 1 of 2)
			Every Monday (week 2 of 2) *etc*
1 day every 4 weeks (4-weekly)	28	28	Every Sunday (week 1 of 4)
			Every Sunday (week 2 of 4)
			Every Sunday (week 3 of 4)
			Every Sunday (week 4 of 4) *etc*

## References

[b1] Arnold P. Losses of nitrous oxide from soil Journal of Soil Science 5, 116–128 (1954).

[b2] Denmead O. Chamber systems for measuring nitrous oxide emission from soils in the field. Soil Sc. Soc. Am. J. 43, 89–95 (1979).

[b3] Hutchinson G. & Mosier A. Improved Soil cover method for field measurement of nitrous oxide fluxes. Soil Sc. Soc. Am. J. 45, 311–316 (1981).

[b4] Ravishankara A., Daniel J. & Portmann R. Nitrous oxide (N_2_O): the dominant ozone-depleting substance emitted in the 21st century. Science 326, 123–125 (2009). 10.1126/science.1176985 19713491

[b5] Crutzen P. J. in Denitrification, nitrification, and atmospheric nitrous oxide (ed Delwiche C C ) 17–44 (John Wiley & Sons, 1981).

[b6] Butterbach-Bahl K., Baggs E., Dannenmann M., Kiese R. & Zechmeister-Boltenstern S. Nitrous oxide emissions from soils: how well do we understand the processes and their controls? Phil Trans R Soc B 368, 20130122 (2013). 10.1098/rstb.2013.0122 PMC3682742 23713120

[b7] Pihlatie M. *et al.* Comparison of static chambers to measure CH_4_ emissions from soils. Agr. Forest. Meteorol. 171–172, 124–136 (2013).

[b8] Denmead O. T. Approaches to measuring fluxes of methane and nitrous oxide between landscapes and the atmosphere. Plant Soil 309 309, 5–24 (2008).

[b9] Parkin T. B. Effect of sampling frequency on estimates of cumulative nitrous oxide emissions. J. Environ. Qual. 37, 1390–1395 (2008). 10.2134/jeq2007.0333 18574170

[b10] Barton L. *et al.* Nitrous oxide emissions from a cropped soil in a semiarid climate. Glob. Change Biol. 14, 177–192 (2008).

[b11] Barton L., Murphy D. & Butterbach-Bahl K. Influence of crop rotation and liming on greenhouse gas emissions from a semiarid soil. Agric. Ecosyst. Environ. 167, 23–32 (2013).

[b12] Wolf B. *et al.* Grazing-induced reduction of natural nitrous oxide release from continental steppe. Nature 464, 881–884 (2010). 10.1038/nature08931 20376147

[b13] Morris S., Kimber S., Grace P. & Van Zweiten L. Improving the statistical preparation for measuring soil N_2_O flux by closed chamber. Sci. Total Environ. 465, 166–172 (2013). 10.1016/j.scitotenv.2013.02.032 23490324

[b14] Liu C. *et al.* Nitrous oxide and nitric oxide emissions from an irrigated cotton field in Northern China. Plant Soil 332, 123–134 (2010).

[b15] Smith K. A. & Dobbie K. E. The impact of sampling frequency and sampling times on chamber-based measurements of N_2_O emissions from fertilized soils. Global Change Biol. 7, 933–945 (2001).

[b16] van der Weeden T., Clough T. & Styles T. Using near-continuous measurements of N_2_O emission from urine-affected soil to guide manual gas sampling regimes. New Zeal. J. Agr. Res. 56, 60–76 (2013).

[b17] Reeves S. & Wang W. Optimum sampling time and frequency for measuring N_2_O emissions from a rain-fed cereal cropping system. Science of The Total Environment 530–531, 219–226, doi: 10.1016/j.scitotenv.2015.05.117 (2015). 26046430

[b18] Flessa H. *et al.* N_2_O and CH_4_ fluxes in potato fields: automated measurement, management effects and temporal variation. Geoderma 105, 307–325 (2002).

[b19] Rowlings D., Grace P., Scheer C. & Kiese R. Influence of nitrogen fertiliser application and timing on greenhousegas emissions from a lychee (Litchi chinensis) orchard in humid subtropical Australia. Agric. Ecosyst. Environ. 179, 168–178 (2013).

[b20] Stehfest E. & Bouwman L. N_2_O and NO emission from agricultural fields and soils under natural vegetation: summarizing available measurement data and modeling of global annual emissions. Nutr. Cycl. Agroecosystem 74, 207–228 (2006).

[b21] de Klein C. & Harvey M. Nitrous oxide chamber methodology guidelines. (Ministry for Primary Industries, 2012).

[b22] Breuer L., Papen H. & Butterbach-Bahl K. N_2_O emission from tropical forest soils of Australia. J. Geophys. Res. 105 26, 353–326,367 (2000).

[b23] Kiese R., Hewett B., Graham A. & Butterbach-Bahl K. Seasonal variability of N_2_O emissions and CH_4_ uptake by tropical rainforest soils of Queensland, Australia. Global Biogeochem. Cycles 17, 1043 (2003).

[b24] Efron B. Computers and the theory of statistics: Thinking the unthinkable. SIAM Review 21, 460–480 (1979).

[b25] Efron B. & Gong G. A leisurely look at the bootstrap, the jackknife, and cross-validation. Am. Stat. 37, 36–48 (1983).

[b26] Li Y., Barton L. & Chen D. Simulating response of N_2_O emissions to fertiliser N application and climatic variability from a rain-fed and wheat-cropped soil in Western Australia. J. Sci. Food Agric. 92, 1130–1143 (2012). 10.1002/jsfa.4643 21953483

[b27] Barton L., Murphy D. V., Kiese R. & Butterbach-Bahl K. Soil nitrous oxide and methane fluxes are low from a bioenergy crop (canola) grown in a semiarid climate. Glob. Change Biol. Bioenergy 2, 1–15 (2010).

[b28] Barton L., Butterbach-Bahl K., Kiese R. & Murphy D. Nitrous oxide fluxes from a grain-legume crop (narrow-leafed lupin) grown in a semiarid climate. Glob. Change Biol. 17, 1153–1166 (2011).

[b29] Papen H. & Butterbach-Bahl K. A 3-year continuous record of nitrogen trace gas fluxes from untreated and limed soil of a N-saturated spruce and beech forest ecosystem in Germany: 1. N_2_O emissions. J. Geophys. Res. 104, 18, 487–418,503 (1999).

[b30] Wu X. *et al.* Environmental controls over soil–atmosphere exchange of N_2_O, NO and CO_2_ in a temperate Norway spruce forest. Global Biogeochem. Cycles 24, 773–787 (2010).

[b31] Scheer C., Grace P., Rowlings D. & Payero J. Nitrous oxide emissions from irrigated wheat in Australia: impact of irrigation management. Plant Soil 359, 351–362 (2012).

[b32] Scheer C., Grace P., Rowlings D. & Payero J. Soil N_2_O and CO_2_ emissions from cotton in Australia under varying irrigation management. Nutr. Cycl. Agroecosystem 95, 43–56 (2013).

[b33] Rowlings D., Grace P., Scheer C. & Liu S. Rainfall variability drives interannual variation in N_2_O emissions from a humid, subtropical pasture. Sci. Total Environ. 512–513, 8–18 (2015). 10.1016/j.scitotenv.2015.01.011 25613765

[b34] Rowlings D., Grace P., Kiese R. & Weier K. Environmental factors controlling temporal and spatial variability in the soil-atmosphere exchange of CO_2_, CH_4_ and N_2_O from an Australian subtropical rainforest. Glob. Change Biol. 18, 726–738 (2012).

