# Supplementary material for: Sampling frequency affects estimates of annual nitrous oxide fluxes
Source: Sci Rep. 2015 Nov 2;5:15912. doi: 10.1038/srep15912 (PMC4629121; doi:10.1038/srep15912)
Supplement: Supplementary Information [file srep15912-s1.doc]

**Sampling frequency affects estimates of annual nitrous oxide fluxes**

L. Barton^1*^, B. Wolf^2^, D. Rowlings^3^, C. Scheer^3^, R. Kiese^2^, P. Grace^3^, K. Stefanova^4^, & K. Butterbach-Bahl^2,5^

^1^Soil Biology and Molecular Ecology Group, School of Earth & Environment (M087), UWA Institute of Agriculture, Faculty of Sciences, The University of Western Australia, 35 Stirling Highway, Crawley, Western Australia 6009, Australia.

^2^Karlsruhe Institute of Technology, Institute for Meteorology and Climate Research Atmospheric Environmental Research (IMK-IFU), Kreuzeckbahnstr. 19, 82467 Garmisch-Partenkirchen, Germany.

^3^Institute for Future Environments, Queensland University of Technology, 2 George Street, Brisbane, Queensland 4000, Australia.

^4^UWA Institute of Agriculture (M089), The University of Western Australia, 35 Stirling Highway, Crawley, Western Australia 6009, Australia.

^5^International Livestock Research Institute (ILRI), Nairobi, Kenya.

# ***Corresponding author at: School of Earth & Environment (M087), The University of Western Australia, 35 Stirling Highway, Crawley WA 6009, Australia. Tel.: +61 8 488 2543; fax: +61 8 488 1050. *E-mail address*: louise.barton@uwa.edu.au

**Supplementary Table 1. Effect of sampling interval on the range of annual N_2_O fluxes (kg N ha^-1^**), and the proportion of the annual flux (%) calculated using a daily sampling interval, for 28 datasets.

| Location^†^ | Dataset | Year | Treatment | Parameter | Sampling interval | | | |
| --- | --- | --- | --- | --- | --- | --- | --- | --- |
|  |  |  |  |  | 3 d **w**eek^-1^ | Weekly | 2-weekly | 4-weekly |
| Wongan Hills, Australia. | 1 | 2009–2010 | Lupin, Lime | Annual N_2_O flux (kg N ha ^1^)  Proportion of annual flux (%)‡ | 0.03–0.07  63–139 | 0.02–0.12  34–256 | 0–0.22  9–470 | 0–0.45  1–935 |
|  | 2 | 2009–2010 | Lupin | Annual N_2_O flux (kg N ha^-1^)  Proportion of annual flux (%) | 0.02–0.06  55–159 | 0–0.11  6–303 | 0–0.22  1–572 | 0–0.43  -10–115 |
|  | 3 | 2009–2010 | Wheat, Lime | Annual N_2_O flux (kg N ha^-1^)  Proportion of annual flux (%) | 0.03–0.07  68–141 | 0.02–0.13  41–251 | 0.02–0.24  34–464 | 0.01–0.45  14–874 |
|  | 4 | 2009–2010 | Wheat | Annual N_2_O flux (kg N ha^-1^)  Proportion of annual flux (%) | 0.03–0.08  61–141 | 0.01–0.14  21–245 | 0.01–0.26  10–461 | 0–0.50  3–893 |
|  | 5 | 2010–2011 | Wheat, Lime | Annual N_2_O flux (kg N ha^-1^)  Proportion of annual flux (%) | 0.04–0.08  64–135 | 0.01–0.12  24–218 | 0–0.17  8–296 | 0–0.19  -6–345 |
|  | 6 | 2010–2011 | Wheat | Annual N_2_O flux (kg N ha^-1^)  Proportion of annual flux (%) | 0.03–0.08  61–137 | 0.01–0.11  17–200 | 0–0.18  7–324 | -0.01–0.21  -27–378 |
|  | 7 | 2010–2011 | Wheat, Lime | Annual N_2_O flux (kg N ha^-1^)  Proportion of annual flux (%) | 0.02–0.04  70–119 | 0.01–0.07  27–201 | 0–0.11  11–348 | -0.01–0.12  -21–377 |
|  | 8 | 2010–2011 | Wheat | Annual N_2_O flux (kg N ha^-1^)  Proportion of annual flux (%) | 0.05–0.08  79–124 | 0.03–0.11  50–162 | 0.02–0.16  32–248 | 0–0.19  4–288 |
| Cunderdin, Australia. | 9 | 2005–2006 | Wheat, no N fertilizer | Annual N_2_O flux (kg N ha^-1^)  Proportion of annual flux^b^ (%) | 0.13–0.18  86–127 | 0.12–0.18  84–123 | 0.10–0.23  72–157 | 0.01–0.32  10–220 |
|  | 10 | 2005–2006 | Wheat, plus N fertilizer | Annual N_2_O flux (kg N ha^-1^)  Proportion of annual flux (%) | 0.15–0.21  92–129 | 0.14–0.22  86–138 | 0.10–0.31  62–189 | 0.04–0.34  23–210 |
|  | 11 | 2006–2007 | Wheat, no N fertilizer | Annual N_2_O flux (kg N ha^-1^)  Proportion of annual flux (%) | 0.07–0.09  87–109 | 0.05–0.11  61–139 | 0.03–0.14  31–173 | -0.10–0.20  -8–249 |
|  | 12 | 2006–2007 | Wheat, plus N fertilizer | Annual N_2_O flux (kg N ha^-1^)  Proportion of annual flux (%) | 0.07–0.13  80–142 | 0.07–0.15  76–163 | 0.04–0.22  44–241 | 0.02–0.26  23–280 |
|  | 13 | 2007–2008 | Canola, no N fertilizer | Annual N_2_O flux (kg N ha^-1^)  Proportion of annual flux (%) | 0.08–0.10  92–106 | 0.07–0.10  83–112 | 0.06–0.14  66–151 | 0.0–0.16  -5–174 |
|  | 14 | 2007–2008 | Canola, plus N fertilizer | Annual N_2_O flux (kg N ha^-1^)  Proportion of annual flux (%) | 0.12–0.14  90–108 | 0.10–0.19  75–141 | 0.08–0.29  61–218 | 0.04–0.45  27–336 |
|  | 15 | 2008–2009 | Lupin, no N fertilizer | Annual N_2_O flux (kg N ha^-1^)  Proportion of annual flux (%) | 0.10–0.14  84–114 | 0.09–0.20  74–165 | 0.06–0.27  47–218 | 0–0.42  4–339 |
|  | 16 | 2008–2009 | Bare, no N fertilizer | Annual N_2_O flux (kg N ha^-1^)  Proportion of annual flux (%) | 0.08–0.31  60–231 | 0.07–0.46  51–345 | 0.05–0.51  40–384 | 0.03–0.92  21–693 |

**Supplementary Table 1 (continued). Effect of sampling interval on the range of annual N_2_O fluxes (kg N ha^-1^), and the proportion of the annual flux (%) calculated using a daily sampling interval, for 28 datasets.**

| Location^†^ | Dataset | Year | Treatment | Parameter | Sampling interval | | | |
| --- | --- | --- | --- | --- | --- | --- | --- | --- |
|  |  |  |  |  | 3 d **w**eek^-1^ | Weekly | 2-weekly | 4-weekly |
| Xilin, Inner Mongolia. | 17 | 2007–2008 | Grassland, not grazed | Annual N_2_O flux (kg N ha^-1^)  Proportion of annual flux (%)^‡^ | 0.21–0.23  98–108 | 0.17–0.26  78–123 | 0.13–0.37  63–173 | -0.01–0.61  -3–287 |
| Höglwald, Germany. | 18 | 1996 | Spruce and beech Forest | Annual N_2_O flux (kg N ha^-1^)  Proportion of annual flux (%) | 2.40–2.58  97–105 | 2.90–2.16  88–118 | 1.93–2.97  78–120 | 1.49–3.84  60–156 |
|  | 19 | 1997 | Spruce and beech Forest | Annual N_2_O flux (kg N ha^-1^)  Proportion of annual flux (%) | 0.56–0.59  96–100 | 0.54–0.61  92–105 | 0.34–0.67  59–115 | 0.24–0.64  41–110 |
| Kingsthorpe, Australia. | 20 | 2009–2010 | Wheat-cotton, N fertilized, irrigated (low) | Annual N_2_O flux (kg N ha^-1^)  Proportion of annual flux (%) | 1.28–3.06  44–105 | 1.20–3.36  41–115 | 0.94–4.11  32–140 | 0.68–5.31  23–182 |
|  | 21 | 2009–2010 | Wheat-cotton, N fertilized, irrigated (medium) | Annual N_2_O flux (kg N ha^-1^)  Proportion of annual flux (%) | 1.07–2.71  41–104 | 0.98–3.45  38–132 | 0.74–4.68  28–179 | 0.38–6.36  14–244 |
|  | 22 | 2009–2010 | Wheat-cotton, N fertilized, irrigated (high) | Annual N_2_O flux (kg N ha^-1^)  Proportion of annual flux (%) | 1.36–3.12  46–105 | 1.17–3.31  39–111 | 0.96–3.68  32–124 | 0.44–4.55  15–154 |
| Mooloolah Valley, Australia. | 23 | 2007–2008 | Pasture | Annual N_2_O flux (kg N ha^-1^)  Proportion of annual flux (%) | 1.38–1.69  86–106 | 1.11–2.02  69–126 | 0.56–1.83  35–114 | 0.29–2.38  18–149 |
|  | 24 | 2008–2009 | Pasture | Annual N_2_O flux (kg N ha^-1^)  Proportion of annual flux (%) | 1.93–2.23  91–105 | 1.71–3.13  81–148 | 1.35–3.31  64–156 | 0.96–4.51  45–213 |
|  | 25 | 2008–2009 | Rainforest | Annual N_2_O flux (kg N ha^-1^)  Proportion of annual flux (%) | 0.46–0.48  97–101 | 0.41–0.52  85–110 | 0.37–0.53  79–112 | 0.30–0.51  62–106 |
|  | 26 | 2007–2008 | Tree crop (lychee) | Annual N_2_O flux (kg N ha^-1^)  Proportion of annual flux (%) | 1.58–1.68  94–100 | 1.35–1.78  80–106 | 0.84–2.15  50–128 | 0.47–2.30  28–136 |
|  | 27 | 2008–2009 | Tree crop (lychee) | Annual N_2_O flux (kg N ha^-1^)  Proportion of annual flux (%) | 7.56–8.49  93–102 | 6.37–8.52  78–105 | 5.43–10.07  67–124 | 3.29–14.31  40–176 |
| Bellenden Ker, Australia. | 28 | 2001–2002 | Rainforest | Annual N_2_O flux (kg N ha^-1^)  Proportion of annual flux (%) | 1.14–1.18  98–102 | 1.08–1.19  93–103 | 0.84–1.25  72–107 | 0.66–1.43  57–123 |

^†^Cunderdin, 31°36′S, 117°13′E; Wongan Hills, 30°89′S, Höglwald 116°72′E; 48°30′N, 11°10′E; Xilin 43° 33′ N, 116° 42.3′ E; Bellenden Ker, 17°16′S, 145°54′E; Kingsthorpe, 27°30′S, 151°46′E; Mooloolah Valley 26.75° S, 152.93° E

^‡^The mean annual flux estimate from each sampling frequency compared to the overall mean annual flux calculated from all the daily fluxes.
